# Suicidal acid ingestion leading to gastric outlet obstruction treated by early definitive surgery—case report

**DOI:** 10.1093/jscr/rjab027

**Published:** 2021-02-19

**Authors:** Mutlaq Almalki, Waed Yaseen, Shatha Althobaiti

**Affiliations:** Alnoor Specialist Hospital, General Surgery Department, Makkah, Saudi Arabia; Alnoor Specialist Hospital, General Surgery Department, Makkah, Saudi Arabia; Alnoor Specialist Hospital, General Surgery Department, Makkah, Saudi Arabia

## Abstract

Chemical ingestions can cause acute injury to the oesophagus, stomach, pylorus, duodenum and sometimes other organs after ingestion of corrosives, but it may be as late as 1 year after ingestion. A 30-year-old male patient presented to the emergency department with sudden epigastric abdominal pain after flash material ingestion. Computed tomography of abdomen showed signs of small bowel obstruction associated with segmental small bowel ischaemic changes. Postoperatively, patient developed an intolerance to oral intake with upper gastrointestinal scope showing sever stricture at the distal gastric lumen and pylorus. The patient was taken to the operation where gastrojejunostomy and brown procedure was done. Corrosive gastric injury treatment depends on the degree of gastric involvement, related oesophageal strictures and the patient’s general health. Early surgery offers very satisfactory and physiological results, whereas avoiding gastric resection or bypass provides very satisfactory and physiological outcomes.

## INTRODUCTION

Any substance capable of causing tissue damage is known as ‘corrosive’. The most commonly ingested corrosive chemicals are strong acids and bases (pH < 2 or >12), which can rapidly penetrate the various layers of the oesophagus, but other chemical substances can also cause such injuries, for example, concentrated acetic acid. Chemical ingestions can cause acute injury to the oesophagus, stomach, pylorus, duodenum and sometimes other organs. World Health Organization reported that the approximate global incidence of corrosive ingestions is 110/100 000 individuals per-year. Worldwide corrosive ingestion mortality was 310 000 persons in 2004 or 4.8/100 000 population per-year [1]. Acid ingestion induces pyloric spasm and causes acid pooling in the pyloric region, thus creating more contact time for acid to cause mucosal oedema and inflammation. The average duration of progression to gastric outlet obstruction is ~4–6 weeks after ingestion of corrosives, but it may be as late as 1 year after ingestion [2]. Other upper gastrointestinal (GI) complications noted were bleeding, perforation and stricture formation [1].

## CASE PRESENTATION

**Figure 1:**
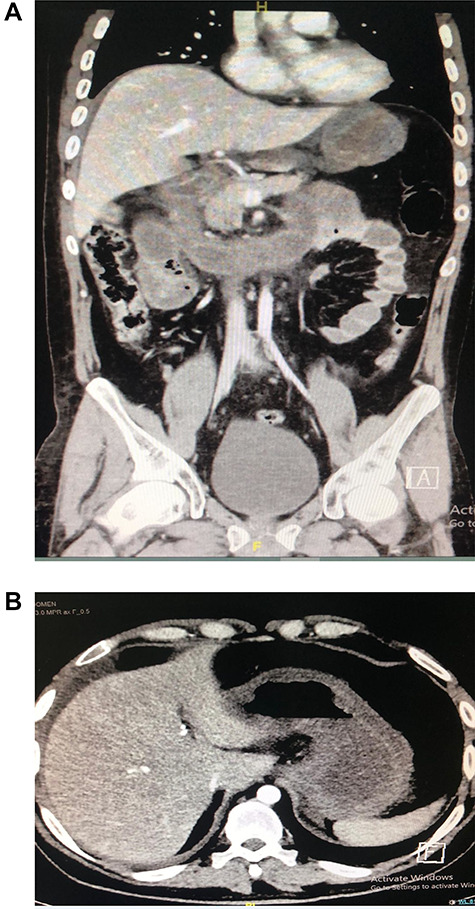
(**A** and **B**). CT showing segmental dilatation of the small bowel with diffuse circumferential wall thickening and suspected tow areas of transnational zones.

A 30-year-old male patient presented to emergency department with epigastric abdominal pain for 30 minutes after flash material ingestion. He was conscious, oriented, with vital signs remarkable for tachycardia; abdominal examination was soft with epigastric tenderness. Lab investigations were haemoglobin: 17, white blood cell: 19, normal renal profile and amylase. Computed tomography (CT) abdomen was done and showed signs of small bowel obstruction associated with segmental small bowel ischaemic changes (Fig. 1a and b).

**Figure 2:**
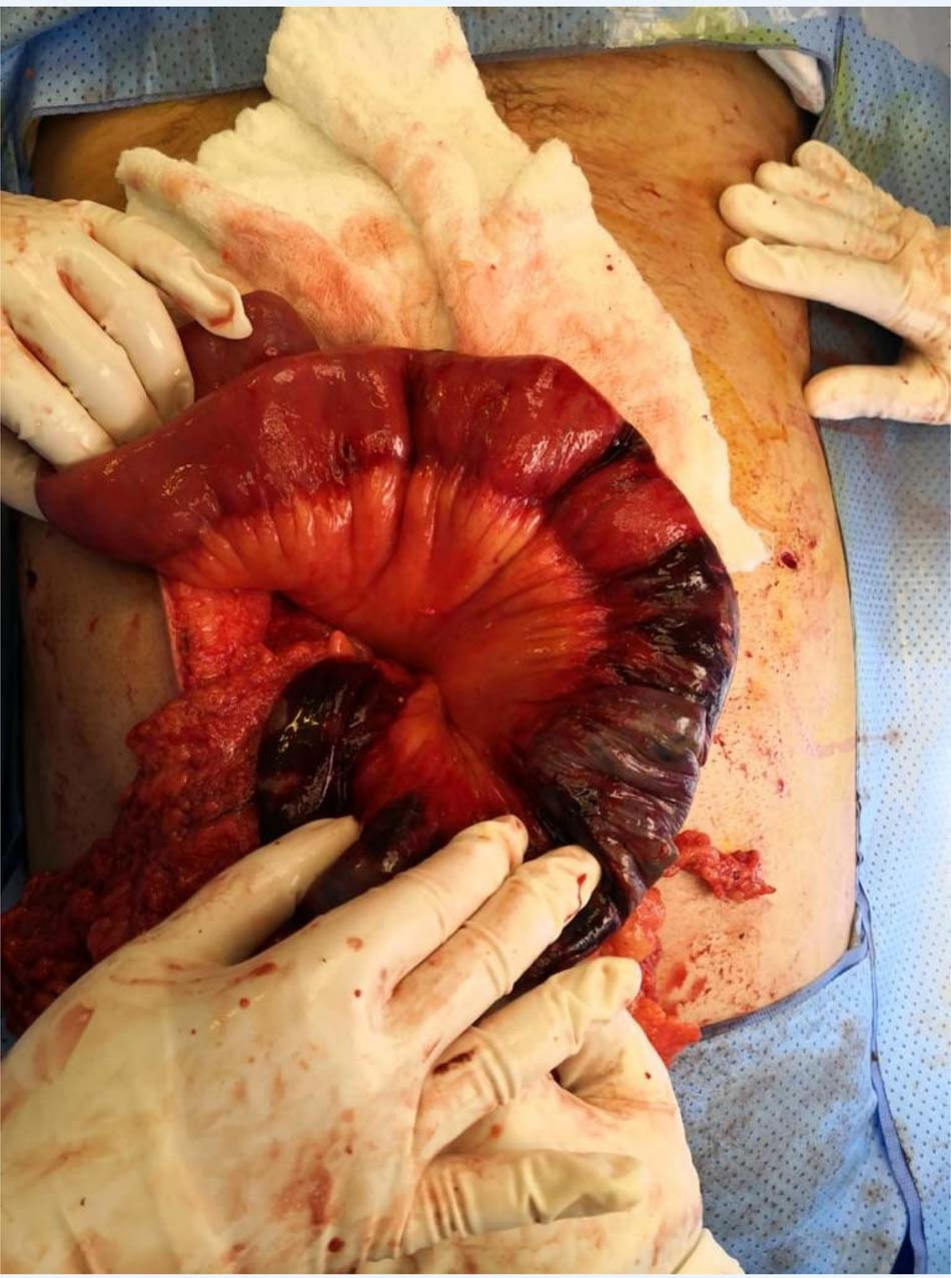
Ischaemic patches at the fourth and proximal 70 cm of jejunum.

The patient was taken for diagnostic laparoscopy converted to open; findings were ischaemic patches at the fourth and proximal 70 cm of jejunum (Fig. 2); resection was done with duodenal–jejunal anastomosis and feeding jejunostomy. The patient was doing fine postoperatively and tolerating jejunostomy feeding. After a while, he developed difficulty in tolerating oral feeding; gastrografin study was unremarkable. The patient underwent esophagogastroduodenoscopy (EGD) that showed ulcerations were detected at mid oesophagus. Cardia was patent and the scope passed easy to stomach, which had severe pangastritis associated with friable mucosa and multiple shallow ulcerations with narrow pyloric ring, but the scope passed and showed friable mucosa with erosions distal to pyloric ring. Thirteen days later gastrografin study was done as he still cannot tolerate oral feeding; it revealed sever stricture at the distal gastric lumen and pylorus.

He underwent multiple attempts of dilatation by upper GI endoscopy; all failed to dilate pyloric stenosis. Decision was made to take patient to second laparotomy with findings of: adhesions all over the abdomen, thick friable stomach and bowel. Gastrojejunostomy and brown procedure performed. Postoperatively, the patient had wound infection with culture showed *Klebsiella pneumoniae*, which was treated conservatively followed by smooth hospital course with no major event.

## DISCUSSION

Acid and alkali corrosive ingestion was more commonly reported in developing countries. Ingestion of corrosives in adults is more often suicidal in intent and therefore tends to be more serious [3]. A significant number of patients have predominant gastric injury leading to gastric strictures. Patients experience pain, vomiting, rapid loss of weight and malnutrition. Almost every patient needs operation [4]. The extent of damage in the oesophagus and stomach depends on the agent, amount ingested and duration of contact. Acids are easily expelled from the oesophagus to the stomach and, due to chemical irritation, induce pylorospasm. Acids having a longer duration of contact with the gastric mucosa cause coagulative necrosis resulting gastric strictures [5].

Corrosive gastric injury has been categorized into five forms by Ananthakrishnan *et al*. [5], based on EGD and barium assessment. Type I—Short ring stricture of the stomach within 1–2 cm of the pylorus. Type-II—Stricture extending proximally up to the antrum. Type-III—Mid-gastric stricture involving the body and sparing the proximal and distal parts of the stomach. Type-IV—Diffuse gastric involvement like linitis plastica. Type-V—Gastric stricture associated with a stricture in the first part of the duodenum.

Nonsurgical management by endoscopic dilatation is unsatisfactory in most of the times. Short-segment gastric stricture can be managed by endoscopic dilatation, but the majority of the patients need surgical intervention [5].

Surgical treatment of corrosive gastric stricture is typically postponed in order to restore poor general health, malnutrition and inflammation of the stomach, so that the extent of scarring becomes apparent [5].

As in our case many patients need feeding jejunostomy initially as an alternative route of feeding. There is no clear consensus of what is the ‘early’ definitive operative timing [6]. Hwang *et al*. [7] and Ray and Adak [4] compared early definitive surgery within 1–4 months after injury with delayed surgery and concluded that early definitive treatment can give a better quality of life to the patients. According to literature surgical options for gastric stricture due to corrosive ingestion can be classified to three categories: (i) resection in the form of Billroth I or II gastrectomy, which is reported in many series [5, 8, 9]; (ii) bypass the gastric stricture in the form of gastrojejunostomy either loop or Roux-en-Y [8–10]; (iii) strictureplasty for short-segment strictures, which is a simple operation and can be done early in the majority of cases [5].

In our case, the patient was taken for laparoscopic exploration as with finding of ischaemic fourth part of duodenum along with the proximal 70 cm of jejunum; so we proceeded with resection and duodenojejunostomy with the creation of jejunostomy feeding tube. After follow-up with endoscopy, we found that the patient has gastric outlet obstruction, and so we proceeded with diversion with gastrojejunostomy with brown procedure.

## CONCLUSION

We could summarize the management of gastric outlet obstruction due to corrosive ingestion to two distinct stages: the initial one is the feeding jejunostomy stage aiming to improve patient’s nutritional status followed by the definitive surgery stage, of which have been categorized to three different options according to literature, but further studies and longer follow-up intervals are needed to standardize the management of such cases.
